# Spirit of the British and American Periodicals, &c.

**Published:** 1839

**Authors:** 


### Spirit of the British and American Periodicals, &c.

---

### On the Effects of Mental Attention on Bodily Organs. By Dr. Holland.

This subject occupies the 5th Chapter of Dr. Holland's recent publication. It is a curious investigation, and has by no means been exhausted by physiological or practical writers. The influence of the will on the voluntary muscles, and of passions of the mind on the various involuntary functions, has long been familiar to medical observers; but Dr. Holland asks:—

"Among the phenomena which attest the various influence of the mind on the bodily organs, has sufficient notice been taken of those peculiar effects which depend on the simple act of concentrating the attention separately upon them?"

The effects of this "attention," or, as Dr. H. would prefer, this "direction of consciousness," to the heart and respiratory apparatus, are rather ambiguous, since the said organs are affected, more or less, by almost every emotion that passes through the mind, and the act of "attention" can, with difficulty be separated from these.

"Yet there is cause to believe the action of the heart quickened, or otherwise disturbed, by the mere direction of consciousness to it, without any emotion or anxiety. On occasions where its beats are audible, observation closely applied will give proof of this. And where there is liability to irregular pulsation, this is seemingly brought on or increased by the simple effort of attention, even though no obvious emotion be present."

We apprehend that this attention to the action of one's own heart is seldom exerted without some *cause* for emotion of mind, as apprehension of disease, &c. and consequently where there cannot be an exact deduction drawn. If a medical or any other man tries the pulse by way of experiment, he will very soon find (by making) what he is in search of. There are other organs and functions, however, where the effects of attention are less equivocal. Thus the act of deglutition is frequently rendered more difficult by our attention being fixed on that process. Where there is any impediment of speech, there is increase of difficulty in proportion to the attention of the individual to the organs of articulation. There is some truth, but we think also some fallacy, in the following observations.

"A similar direction of consciousness to the region of the stomach creates in this part a sense of weight, oppression, or other less definite uneasiness; and when the stomach is full, appears greatly to disturb the due digestion of the food. It is remarkable how instantly under such circumstances the effect comes on; a fact readily attested by experiment which every one may make for himself. This part of the body is well known to be singularly under the influence of all mental emotions; and it is difficult, as we have seen, so far to individualize or simplify the act of attention as to remove it wholly out of this class. The symptoms of the dyspeptic patient are doubtless much aggravated by the constant and earnest direction of the mind to the digestive organs, and the functions going on in them. Feelings of nausea may be produced, or greatly increased, in this way; and are often suddenly relieved by the attention being diverted to other objects. The proofs of this are frequent and familiar, especially in the curious phenomena of sea-sickness.—Further, the state and action of the bowels are much influenced by the same cause. Sensations occur of which we were not before conscious, and the actions of the lower bowels in particular are obviously excited and quickened. Such also is the case with the bladder; which is solicited to act, when not otherwise disposed, by attention directed to it; the cessation of this removing at once all present sense of need."

The salivary glands are liable to be affected in their secretions by attention being intently directed to them; and "in the gums, the sensation created by attention given to them may rise almost to pain." This, we confess, staggers us a little; but we suppose it must be all right. It is clear that all these experiments must be self-executed:—they cannot be done by proxy. Dr. Holland must have experienced all these curious sensations in his own person; and it is evident that his acute—we had almost said *morbid sensibilities*, would render him an excellent subject for animal magnetism. Let the worthy doctor beware how he comes within the range of Dr. Elliotson's influence. The whole paper is, in fact, an *auto-mesmeric* disquisition, or rather series of experiments, from beginning to end. We shall cull a few more examples. "The feelings produced in the tongue in like way are peculiar and well marked. Or a single limb, or portion even of a limb, may be taken for experiment; and a peculiar sense of weight and restlessness, approaching even to cramp, be produced by urging the attention expressly upon it." "Sensations of heat or cold, or other more vague feelings, on the surface of the body, may readily be created in a similar way." "No one can direct his consciousness to the organs of seeing, hearing, or of taste, without becoming aware of some changes in their state, from the mind thus by effort applied to them." "The attention concentrated, for so by effort of will it may be, on the head or sensorium, gives certain feelings of tension and uneasiness, caused possibly by some change in the circulation of the part; though it may be an effect, wholly inapproachable by proof, on the nervous system itself. Persistence in this effort, which is seldom indeed beyond a short time without confusion, produces results of much more complex nature, and scarcely to be defined by any common terms of language. It is in fact the case of the mind turned inwards upon the organs which most closely minister to its own operations."

Dr. H. explains the various anomalous feelings of the hypochondriac on this principle—such as palpitation, flatulence, hurried breathing, irritable bladder, &c. &c.

"In chorea, hysteria, and diseases where disordered nervous actions occur, the same principle is more or less concerned; but without equally distinct intervention of consciousness and the will, as in most of the instances already cited. Yet in hysteria, the instances are frequent of attacks brought on by the mere expectation of them; or by imitation; or occasionally even by a sort of morbid solicitation of the organs to these singular actions."

Dr. H. has known asthma brought on by the sight of another person labouring under an attack of the same complaint. But what we have already quoted will have prepared the reader for the following declaration of AUTO-MESMERISM.

"I doubt not that certain of the results of animal magnetism are to be explained by reference to these facts. In some instances, where I have seen the magnetizer perform his operations on a limb, and then inquire as to the feelings created in it, the sensations expressed by the patient were such as might readily be created by the solicitation of the question, especially under the nervous condition already induced. The same concentration of consciousness on the head or præcordia, when the mind is under this excitement, creates effects more remarkable and various; and in hysterical habits such as may appear to belong to a more mysterious cause. Other illustrations to the same effect will readily occur to those who have witnessed experiments thus directed, and will apply to this source for their solution."

It is true that no great compliment is here paid to animal magnetism, since the effects produced by that *science* (?) are all traced to the imagination—not the imagination of the magnetiser, but of the magnetized. The former, however, will avail themselves of Dr. Holland's admissions, and argue, not very unreasonably, that if a man can produce miracles in his own person, he may do the same in the person of another. But we very much suspect that our author has suffered himself to be deceived, or rather that he is of that peculiar nervous temperament in which *imagination* occasionally obscures the judgment. We have tried, over and over again to induce some of those sympathies by the force of consciousness directed to different parts, but, in no instance, with the slightest success. However, we may be made of stuff too sceptical for such fine operations, since none of the magnetisers could ever make the slightest impression on us by the most energetic manipulations. In making these observations we have no intention of classing the estimable author of this volume with the believers, or rather the make-believers, of animal magnetism. What Dr. Holland has stated, we have no doubt he has experienced in his own person, and therefore believed; but we reiterate our suspicions that the same results will rarely follow the same causes in other persons.

